# Humanistic care competency among standardized residency training physicians and refresher doctor in prenatal ultrasound: current status and influencing factors

**DOI:** 10.3389/fmed.2026.1822143

**Published:** 2026-04-23

**Authors:** Li Xu, Yue Zhang, Junyan Ma, Yingming Zheng, Peiqiong Chen, Junmei Wang

**Affiliations:** 1 Department of Ultrasound, Women’s Hospital, Zhejiang University School of Medicine, Hangzhou, Zhejiang, China; 2 Women’s Reproductive Health Laboratory of Zhejiang Province, Women’s Hospital, Zhejiang University School of Medicine, Hangzhou, Zhejiang, China; 3 Department of Reproductive Endocrinology, Women’s Hospital, Zhejiang University School of Medicine, Hangzhou, Zhejiang, China; 4 Department of Gynecology, Women’s Hospital, Zhejiang University School of Medicine, Hangzhou, Zhejiang, China

**Keywords:** humanistic care, prenatal ultrasound, professional competency, refresher doctor, standardized residency training

## Abstract

**Objective:**

To investigate the current status of humanistic care competency among standardized residency training (SRT) physicians and refresher doctor in prenatal ultrasound and to identify its associated influencing factors.

**Methods:**

Physicians and visiting physicians undergoing prenatal ultrasound SRT and refresher doctor at our institution between January 2022 and December 2025 were enrolled. Data were collected via a structured self-administered questionnaire. Univariate analysis and multiple linear regression were applied to identify factors independently associated with humanistic care competency. Multivariable models were adjusted for major confounders including baseline empathy, workload, and previous communication training.

**Results:**

A total of 160 participants were included in the final analysis. The mean total Caring Ability Inventory (CAI) score was 189.66 ± 20.56, with the Understanding (U) dimension yielding the highest mean subscale score (74.54 ± 10.31). CAI scores differed significantly according to the frequency of humanities-related training received during schooling and the frequency of active self-directed learning of humanistic care knowledge (both *p* < 0.05). The mean Family APGAR, Perceived Social Support Scale (PSSS), and Jefferson Scale of Empathy–Health Professionals (JSE-HP) scores were 7.54 ± 1.21, 53.98 ± 10.68, and 90.88 ± 13.97, respectively. Spearman correlation analysis revealed that frequent active self-directed learning of humanistic care knowledge, receiving ≥3 sessions of humanities-related training during schooling, and higher JSE-HP, APGAR, and PSSS scores were all positively correlated with total CAI score and each dimension score (all *p* < 0.05). Multiple linear regression confirmed these five variables as independent positive predictors of humanistic care competency (all *p* < 0.05).

**Conclusion:**

The mean total CAI score among prenatal ultrasound SRT and refresher doctor physicians and visiting physicians at our institution was 189.66 ± 20.56. Frequent active self-directed learning of humanistic care knowledge, receiving ≥3 sessions of humanities-related training during schooling, and higher JSE-HP, APGAR, and PSSS scores were identified as positive predictors of humanistic care competency. Strengthening targeted training programs and providing systematic guidance are recommended to enhance humanistic care competency among SRT and refresher doctor physicians.

## 1 Introduction

Humanistic care competence is a modern literacy centered on respecting individuals’ subjective status, physical and mental health, and dignity, manifested as understanding, empathy, compassion, and service capabilities toward others. Medical humanistic care represents both an intrinsic requirement of the practice of medicine and a cornerstone of harmonious doctor-patient relationships; it has increasingly emerged as a core determinant of healthcare service quality (1). In obstetric medicine, prenatal ultrasonography serves as a critical safeguard for maternal and fetal health. Beyond its role as a precise diagnostic modality, prenatal ultrasound constitutes an important opportunity for clinicians to establish trust and convey compassion. SRT is a mandatory stage where medical students, after completing their undergraduate education, undergo systematic and standardized training at specialized institutions. This stage is crucial for qualified clinicians to master clinical skills.

Continuing medical education (CME) is a pivotal component of the medical education system, effectively facilitating the continuous updating of physicians’ knowledge and the enhancement of their skills. Among its components, sending refresher doctor to advanced clinical training bases is a critical aspect of CME. This approach enables ultrasound specialists to conduct in-depth research in specific fields, master cutting-edge technologies, and promote the dissemination of advanced techniques to primary care settings. Such efforts are of significant importance in alleviating disparities in healthcare resource allocation and improving the patient experience. As the future backbone of obstetric ultrasound diagnosis, SRT and refresher doctor physicians exert a direct influence on pregnant women’s examination experience, patient satisfaction, and the quality of the doctor-patient relationship through their humanistic care competency (2). Excellent humanistic care competency enables pregnant women—who frequently face uncertainty and anxiety during pregnancy—to feel respected, understood, and supported, thereby facilitating cooperation during examinations and ultimately improving diagnostic accuracy and efficacy. Nevertheless, in the current medical education environment, curricula tend to prioritize the acquisition of professional knowledge and technical skills, while the cultivation of humanistic care competency is often insufficiently emphasized (3). During the SRT and refresher doctor period, physicians face heavy clinical workloads and considerable learning demands, leaving limited capacity to attend to the humanistic needs of patients. Furthermore, existing SRT and refresher doctor systems lack well-developed evaluation frameworks for humanistic care competency, and the training provided is often neither systematic nor sufficiently targeted (4). Meanwhile, current research on the humanistic care capabilities of prenatal ultrasound SRT physicians and resident physicians remains insufficient.

Elucidating the current status of humanistic care competency among prenatal ultrasound SRT and refresher doctor physicians and conducting a rigorous analysis of its influencing factors are therefore of critical importance for optimizing SRT and refresher doctor programs and enhancing physicians’ comprehensive professional qualities. Such efforts would not only improve the quality of prenatal ultrasound services but also propel obstetric medical care toward greater patient-centeredness and individualization (5). Accordingly, the present study aimed to characterize the humanistic care competency of prenatal ultrasound SRT and refresher doctors, identify its influencing factors, and provide a scientific foundation for developing effective intervention strategies.

## 2 Methods

### 2.1 Study participants

This study adopted the validated Caring Ability Inventory (CAI) as the framework for measuring humanistic care competency. Sample size was estimated based on the widely applied rule of 5–10 observations per independent variable. Given that this study incorporated 13 key independent variables and anticipated an invalid questionnaire rate of 20%, a formal power analysis was additionally performed (effect size = 0.30, *α* = 0.05, power = 0.80) to validate the rationality of the preliminarily estimated sample size, which was based on the linear regression model (consistent with the study’s core statistical method of multiple linear regression), yielding a minimum required sample size of 82–163. Accounting for practical considerations, a final target of 163 participants was set for questionnaire distribution. Relevant confounding factors, encompassing baseline empathy, workload, and prior communication training, were gathered as covariates for subsequent adjusted analyses.

This study clearly defined and distinguished two groups of physicians at our institution from January 2022 to December 2025 were recruited:

(1) Standardized Residency Training (SRT) physicians: physicians who received formal standardized clinical training after undergraduate medical education.
(2) Refresher doctors: doctors who came for advanced clinical training to improve professional skills in prenatal ultrasound.

Inclusion criteria were as follows: (1) Holding a valid medical practitioner’s license in the People’s Republic of China; (2) Being formally enrolled in the hospital’s prenatal ultrasound SRT or clinical refresher training program, with a participation duration in the program of ≥2 months and <3 years (the hospital implements a 3-year standardized training system for SRT and refresher physicians, with the first month designated as pre-employment professional orientation training); (3) Having a fixed job assignment in the Department of Prenatal Ultrasound and undertaking clinical work related to prenatal ultrasound during the research period; (4) Voluntarily participating in this study and being able to complete the entire questionnaire survey independently.

Exclusion criteria were: (1) Having an accumulative leave of absence (sick leave, maternity leave, paternity leave, personal leave, etc.) of ≥1 month during the research period; (2) Having no fixed assignment in the prenatal ultrasound department or having engaged in clinical work in other relevant clinical departments for a long time during the training period; (3) Having to suspend or terminate the SRT/refresher training program for any reason during the research period; (4) Refusing to provide written informed consent or being unable to complete the questionnaire due to various reasons.

All participants provided written informed consent following a thorough explanation of the study objectives, content, and potential risks, in accordance with the principles of respect for participant autonomy and protection of participant rights. The study protocol was reviewed and approved by the Institutional Ethics Committee of our hospital, ensuring full compliance with established medical ethics principles throughout the research process.

### 2.2 Survey instruments

All the scales selected for this study were standardized instruments that had undergone domestic and international localization validation, with proven reliability and validity. The validated reliability and validity indicators of these scales were directly adopted to establish the basis for measurement:

#### 2.2.1 General information

General demographic data, including age, sex, educational level, and marital status, were collected via the electronic medical records system or direct inquiry.

#### 2.2.2 Humanistic care competency

Humanistic care competency was assessed using the Caring Ability Inventory (CAI), originally developed by Nkongho (6) in 1990 (19) and subsequently translated into Chinese and culturally adapted by Yulian (7) in 2012 (20). Validity testing (content validity and construct validity) for Chinese populations has been completed, and the measurement of humanistic care competence in medical settings has been adapted. The scale has been verified to have good psychometric properties in Chinese clinical and medical education research. In previous validation studies, the Cronbach’s *α* coefficient was 0.87–0.92 for the total scale and 0.75–0.85 for each dimension, indicating excellent internal consistency. The content validity index (CVI) of the scale is 0.89, and the confirmatory factor analysis shows a good fit with the three - dimensional structure (*χ*^2^/df = 2.35, CFI = 0.91, TLI = 0.90, RMSEA = 0.07), supporting good content validity and construct validity.

The CAI comprises 37 items organized across three core dimensions: the Understanding (U) dimension (14 items) which evaluates the ability to empathically recognize patients’ needs and circumstances at both emotional and cognitive levels; the Courage (C) dimension (13 items), which assesses the willingness and capacity to engage with challenging humanistic care situations; and the Patience (P) dimension (10 items), which measures the ability to remain composed, tolerant, and consistently attentive in response to patient needs and challenges. Each item is rated on a 7-point Likert scale (1 = strongly disagree to 7 = strongly agree), yielding a total score ranging from 37 to 259; higher scores indicate greater humanistic care competency. In this study, the Cronbach’s *α* coefficient of the CAI total scale was 0.88, and the coefficients for the U, C, and P dimensions were 0.85, 0.81, and 0.79, respectively. This confirms good internal consistency for the study sample.

#### 2.2.3 Family support

Family support was evaluated using the Family APGAR Index, developed by Smilkstein in 1978 and widely adapted for cross-cultural research, including research on the Chinese population. The scale is a brief and reliable tool for assessing perceived family function and support. It consists of 5 items that measure five core dimensions of family support: Adaptability, Partnership, Growth, Affection, and Resolve.

In Chinese population studies, it has a Cronbach’s *α* coefficient of 0.78–0.85, good test-retest reliability (*r* = 0.82, *p* < 0.001), and content validity (CVI = 0.86). Each item is scored on a 3-point scale (0 = hardly ever, 1 = sometimes, 2 = often), with a total score ranging from 0 to 10. Higher scores indicate a greater degree of perceived family support. In this study, the Cronbach’s *α* coefficient of the scale was 0.80, meeting the psychometric requirements for the current research.

The Family APGAR Index, a classic family support assessment tool that has been validated through years of clinical application, exhibits excellent content validity and criterion-related validity. It is suitable for measuring the perception of family support among diverse populations.

#### 2.2.4 Perceived social support

Perceived social support was measured using the Perceived Social Support Scale (PSSS), developed by Blumenthal et al. (8) and revised for the Chinese population by Duan et al. The revised version is widely used in Chinese medical research.

The scale consists of 12 items, which are divided into three dimensions: family support, friend support, and other support (colleagues, leaders, etc.). Each item is rated on a 7-point Likert scale (1 = strongly disagree to 7 = strongly agree). Total scores range from 12 to 84, with higher scores reflecting greater perceived social support.

The revised Chinese version has been validated to have excellent psychometric properties. The Cronbach’s *α* coefficient of the total scale is 0.89–0.94, the test–retest reliability is 0.85 (*p* < 0.001), and the construct validity is supported by factor analysis (three common factors extracted, cumulative variance contribution rate 78.3%).

In this study, the Cronbach’s *α* coefficient for the PSSS total scale was 0.91, and the coefficients for the three dimensions were 0.89, 0.87, and 0.86, respectively, indicating excellent internal consistency. An *α* coefficient of > 0.90 indicates that the scale has excellent internal consistency reliability, with strong correlations between items across dimensions.

#### 2.2.5 Empathy

Empathy was evaluated using the Jefferson Scale of Empathy–Health Professionals version (JSE-HP) (9), which was developed by Hojat et al. and adapted and validated for Chinese medical professionals by Wang Yanhong et al. The Chinese version of the scale maintains the original three - subscale structure and exhibits favorable psychometric characteristics. An *α* coefficient within the range of 0.70–0.80 suggests that the scale possesses good internal consistency reliability and meets the reliability criteria for measuring empathy in medical populations.

The JSE-HP consists of 20 items distributed across three subscales: perspective taking (7 items, score range: 3–21 points), compassionate care (7 items, score range: 7–49 points), and standing in the patient’s shoes (6 items, score range: 10–70 points), resulting in a total score range of 20–140. Each item is rated on a 7-point Likert scale (1 = strongly disagree to 7 = strongly agree), where higher scores signify greater empathic capacity. In this study, the Cronbach’s *α* coefficient of the JSE-HP total scale was 0.75, and the coefficients for the three subscales were 0.73, 0.71, and 0.69, respectively. This is in line with the psychometric characteristics of the scale in previous medical professional studies and meets the reliability requirements for this research.

### 2.3 Data collection procedure

Prior to data collection, all investigators underwent standardized training covering study objectives, significance, questionnaire completion procedures, and key precautions for participant assistance. Both paper-based and electronic questionnaire formats were used. Questionnaires were completed on-site under investigator supervision, ensuring response completeness and accuracy. Following collection, all questionnaires underwent rigorous quality review; responses failing to meet predefined validity criteria were excluded. A total of 163 questionnaires were distributed, The questionnaire requires clear and legible handwriting for easy recognition and complete responses, and 160 valid questionnaires were retrieved (one questionnaire was incomplete, and two questionnaires had illegible handwriting), yielding an effective response rate of 98.16%.

### 2.4 Statistical analysis

With the total score of the Caring Ability Inventory (CAI) as the dependent variable, the independent variables encompass the frequency of self-directed learning of humanistic care knowledge, the participation frequency in humanistic—related training during the school years, JSE-HP scores, APGAR scores, and PSSS scores. To ensure the validity of the measurement of humanistic care capacity and related variables in this study, the research utilized data distribution tests and validation of the suitability of statistical methods to guarantee the reliability of scale scores and subsequent analyses. All data were analyzed using SPSS version 27.0 (IBM Corporation, Armonk, NY, USA). Normality was evaluated using the Shapiro–Wilk test. Normality tests were conducted on all scale scores (CAI, APGAR, PSSS, JSE-HP), and corresponding statistical description and analysis methods were selected based on the test results.

Continuous variables with a normal distribution are presented as the mean ± standard deviation (*X–* ± s) and compared using the independent—samples *t*-test. Continuous variables with a non-normal distribution are expressed as the median (interquartile range) [M(Q1, Q3)] and analyzed using the Mann–Whitney *U* test. To avoid errors in the interpretation of measurement results caused by data distribution bias and ensure criterion validity, categorical variables are reported as frequencies and percentages and compared using the chi - square test or Fisher’s exact test, as appropriate. Univariate analysis was employed as a pre-screening step for multiple linear regression, with the aim of identifying variables significantly associated with the dependent variable. Taking the total score of the Caring Ability Inventory (CAI) as the dependent variable, the differences in CAI scores among different groups of potential independent variables (including demographic characteristics and learning training characteristics) were compared by the t-test, F -test, or Mann–Whitney U test. Variables with *p* < 0.05 were regarded as statistically significant and included in the subsequent correlation and regression analysis, while irrelevant variables with *p* ≥ 0.05 were excluded to reduce model interference. Univariate and multiple linear regression analyses were carried out to identify factors independently associated with humanistic care competency. In the univariate analysis, initially, the t-test and F-test were used to identify variables significantly associated with CAI scores and eliminate the interference of irrelevant variables. In the multiple linear regression analysis, through the control of multicollinearity among variables, the study verified their independent predictive role in humanistic care capacity. The regression model showed a good fit, with *R*^2^ = 0.413, adjusted *R*^2^ = 0.408, *F* = 18.642, and *p* < 0.001, indicating that the influencing factors had significant explanatory power and ensuring the robustness of the conclusions regarding the determinants of humanistic care capacity.

Spearman correlation analysis was used to quantify associations between variables of interest and verify the correlation between each dimension of CAI and core variables such as empathy, family support, and social support. Spearman rank correlation analysis was performed to explore the direction, strength and statistical significance of the association between variables. A two-tailed *p* < 0.05 was considered statistically significant. Multiple linear regression analysis was conducted to verify the independent predictive effect of core variables on humanistic care competency, with the total CAI score as the dependent variable. The variable selection followed a three-step rigorous process: primary screening by univariate analysis, correlation verification by Spearman rank correlation analysis, and final confirmation by multicollinearity test. Multivariable linear regression was performed with the total CAI score serving as the dependent variable. To mitigate potential confounding factors and enhance the robustness of the model, we adjusted for pre-defined covariates, which included baseline empathy (as measured by the JSE-HP score), work experience, workload, and history of communication training. Other clinically relevant confounding variables, such as age, educational level, and marital status, were also incorporated into the adjustment set. Multicollinearity was examined to guarantee the stability of the model. The model fitting adhered to the standardized procedure: initially, the independent variables were either coded or retained as raw scores based on their types; subsequently, four pre-conditions of multiple linear regression (linear relationship, normality, homogeneity of variance, and no multicollinearity) were tested to guarantee the rationality of the fitting; finally, the Enter method was employed to incorporate all core independent variables into the model at once, and the ordinary least squares method (OLS) was utilized to calculate the model parameters.

The overall fitting effect of the model was evaluated through *R*^2^, adjusted *R*^2^, *F*-value, and *p*-value, and the independent predictive effect of each variable was determined by the *t*-value and *p*-value of the regression coefficient. This finding aligns with the theoretical expectation that ‘humanistic care competence is positively correlated with empathy and social support, indirectly validating the structural validity of the CAI scale in the study population (SRT/refresher doctor).

## 3 Results

### 3.1 CAI scores

**Figure 1:**
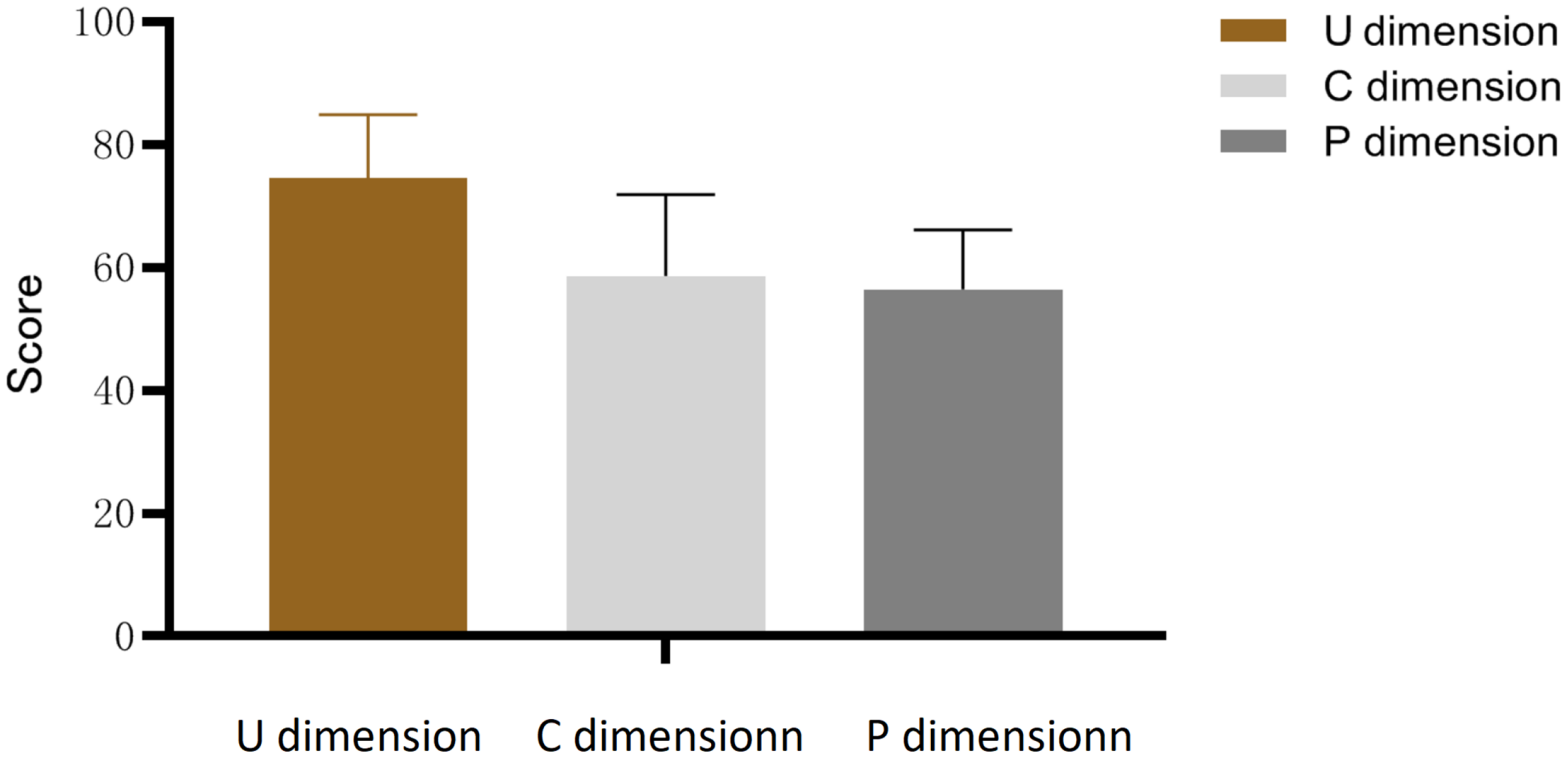
CAI dimension score distribution (*n* = 160).

A total of 160 participants were included in the final analysis. The mean total CAI score was 189.66 ± 20.56. Among the three subscales, the U dimension yielded the highest mean score (74.54 ± 10.31). The distribution of scores across all CAI dimensions is presented in Figure 1.

### 3.2 Univariate analysis of factors influencing humanistic care competency

Significant between-group differences in CAI scores were observed according to the frequency of humanities-related training received during schooling and the frequency of active self-directed learning of humanistic care knowledge (both *p* < 0.05). No significant associations were identified for the remaining variables examined. Detailed results are presented in Table 1.

**Table 1:** Univariate analysis of factors influencing humanistic care competency among prenatal ultrasound SRT and refresher doctor physicians (*n* = 160).

Variable	Category	*n*	CAI (score, $\bar{X}\pm s$ )	*t/F*	*p*
Sex	Female	117	189.76 ± 12.51	0.172	0.864
	Male	43	189.39 ± 10.75		
Age (years)	<30	90	188.84 ± 12.05	0.746	0.457
	≥30	70	190.71 ± 19.49		
Place of residence	Urban	127	190.58 ± 15.35	1.527	0.129
	Rural	33	186.12 ± 13.27		
Educational level	Bachelor’s or below	63	189.11 ± 13.96	0.420	0.675
	Above bachelor’s	97	190.02 ± 13.02		
Work experience (years)	<2	68	188.17 ± 18.16	0.809	0.420
	≥2	92	190.76 ± 21.27		
Marital status	Non-married	39	190.58 ± 12.76	0.464	0.644
	Married	121	189.36 ± 14.74		
Only child	Yes	94	187.94 ± 12.08	1.876	0.063
	No	66	192.11 ± 16.03		
Having children	No	30	193.66 ± 15.81	1.771	0.079
	Yes	130	188.74 ± 13.20		
Humanities-related training during schooling	None	59	170.13 ± 12.61	3.229	0.042
	<3 sessions	43	176.63 ± 16.75		
	≥3 sessions	58	219.19 ± 16.53		
Active self-directed learning of humanistic care knowledge	Rarely	88	171.46 ± 13.96	3.317	0.039
	Sometimes	49	196.34 ± 18.88		
	Frequently	23	245.06 ± 12.73		

### 3.3 APGAR, PSSS, and JSE-HP scores

**Figure 2:**
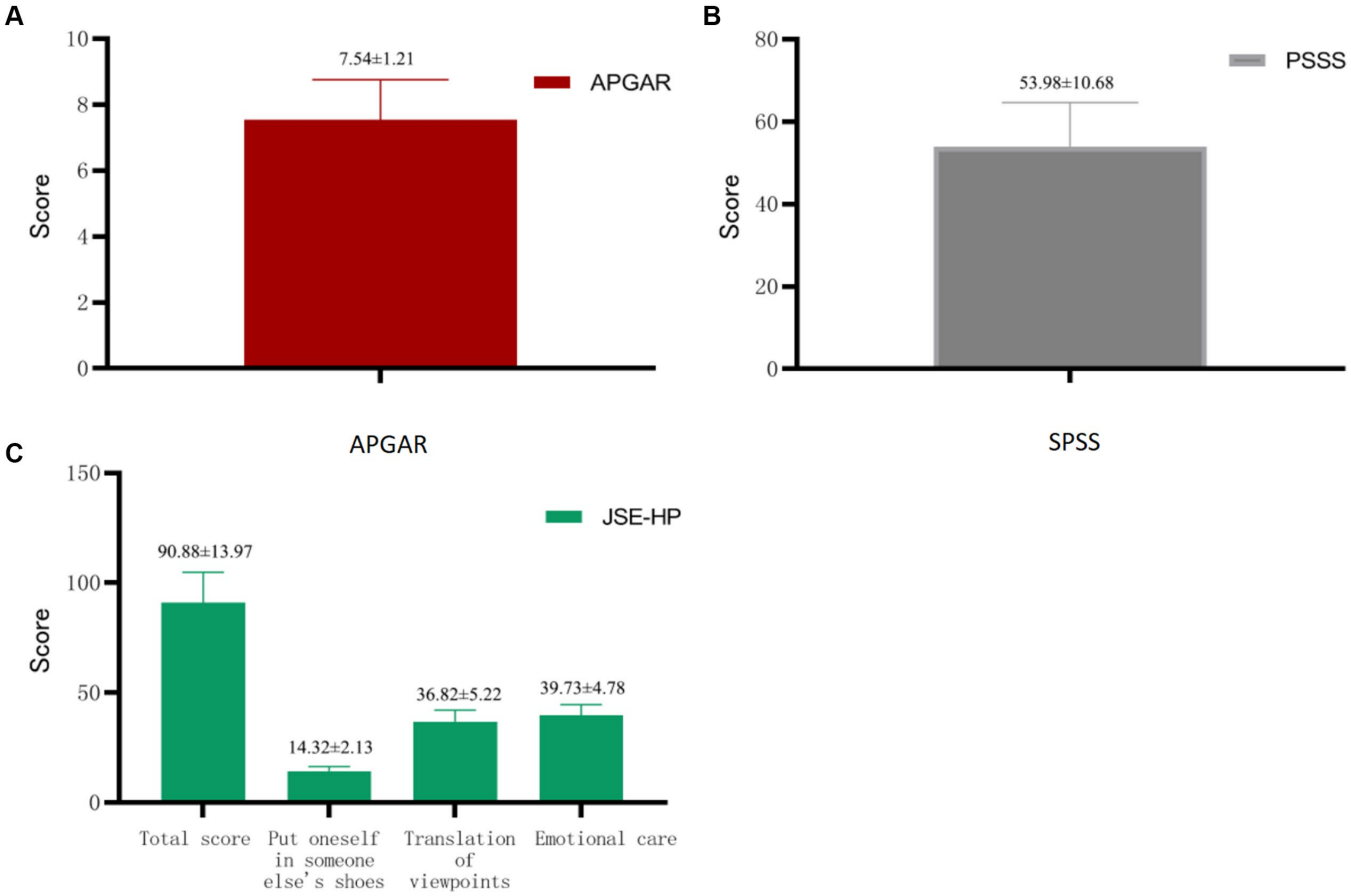
APGAR, PSSS, and JSE-HP score distributions (*n* = 160). From **(A–C)** are APGAR, PSSS, and JSE-HP scores, respectively.

The mean Family APGAR score was 7.54 ± 1.21, the mean PSSS total score was 53.98 ± 10.68, and the mean JSE-HP total score was 90.88 ± 13.97. The score distributions for these three instruments are presented in Figure 2.

### 3.4 Correlation analysis between CAI scores and influencing factors

Based on the Spearman rank correlation analysis method described in Section 2.4, the associations between CAI scores and core factors were analyzed, and the results showed that Spearman correlation analysis was performed to examine associations between each CAI dimension score and the factors identified as significant in the univariate analysis. Frequent active self-directed learning of humanistic care knowledge, receiving ≥3 sessions of humanities-related training during schooling, and higher JSE-HP, APGAR, and PSSS scores were all positively correlated with total CAI score and each dimension score (all *p* < 0.05). Detailed results are presented in Table 2.

**Table 2:** Spearman correlation analysis between CAI dimension scores and influencing factors (*n* = 160).

Factor	U dimension	C dimension	P dimension	Total score
Active self-directed learning of humanistic care knowledge	0.188*	0.192*	0.238*	0.276*
Humanities-related training during schooling (≥3 sessions)	0.216*	0.241*	0.157*	0.224*
JSE-HP score	0.240*	0.177*	0.208*	0.198*
APGAR score	0.181*	0.168*	0.170*	0.196*
PSSS score	0.157*	0.176*	0.142*	0.191*

*p < 0.05.

### 3.5 Multiple linear regression analysis of influencing factors

Multivariable linear regression was conducted with adjustment for key confounding factors, including baseline empathy, work experience, workload, and prior communication training, to guarantee the independence of the effects of predictors. Based on the standardized fitting process of multiple linear regression, using total CAI score as the dependent variable and the statistically significant variables identified through univariate and correlation analyses as independent variables (variable coding scheme detailed in Table 3), multiple linear regression analysis demonstrated that frequent active self-directed learning of humanistic care knowledge, receiving ≥3 sessions of humanities-related training during schooling, and higher JSE-HP, APGAR, and PSSS scores were all independent positive predictors of humanistic care competency among prenatal ultrasound SRT and refresher doctor physicians (all *p* < 0.05). Detailed results are presented in Tables 3, 4.

**Table 3:** Variable coding scheme for multiple linear regression.

Variable	Assignment method
Active self-directed learning of humanistic care knowledge	Rarely = 0, Sometimes = 1, Frequently = 2
Humanities-related training during schooling	None = 0, <3 sessions = 1, ≥3 sessions = 2
JSE-HP score	Raw value entered
APGAR score	Raw value entered
PSSS score	Raw value entered

**Table 4:** Multiple linear regression analysis of factors influencing humanistic care competency (*n* = 160).

Variable	*B* (95%CI)	SE	*β*	*t*	*p*
Constant	30.568 (22.295–38.841)	4.215	–	7.253	<0.001
Active self-directed learning of humanistic care knowledge	2.174 (0.265–4.083)	1.478	0.322	4.548	<0.001
Humanities-related training during schooling (≥3 sessions)	1.032 (0.312–1.752)	0.367	0.156	2.812	0.005
JSE-HP score	0.876 (0.456–1.296)	0.214	0.245	4.093	<0.001
APGAR score	1.726 (0.485–2.967)	0.633	0.143	2.732	0.006
PSSS score	0.731 (0.342–1.120)	0.198	0.213	3.692	<0.001

*R*^2^ = 0.413; adjusted *R*^2^ = 0.408; *F* = 18.642; *p* < 0.001.

Multivariable linear regression was performed with adjustment for key confounders, including baseline empathy, work experience, workload, and history of communication training.

## 4 Discussion

The present study investigated the current status and influencing factors of humanistic care competency among prenatal ultrasound SRT, refresher doctor physicians, and visiting physicians at our institution. By analyzing the questionnaire data from 160 participants using univariate analysis, Spearman correlation analysis, and multiple linear regression, the mean total CAI score was found to be 189.66 ± 20.56. Moreover, frequent active self-directed learning of humanistic care knowledge, receiving ≥3 sessions of humanities-related training during schooling, and higher JSE-HP, APGAR, and PSSS scores were identified as independent positive predictors of humanistic care competency (all *p* < 0.05).

These findings provide quantitative evidence for the cultivation of humanistic care capabilities among medical professionals in obstetric ultrasound practice and also offer targeted directions for optimizing residency training and advanced training systems. The core results are now discussed in conjunction with clinical practice and actual medical education scenarios.

### 4.1 The overall current situation of humanistic care ability among resident physicians and visiting physicians in prenatal ultrasound training programs

The mean total CAI score of 189.66 ± 20.56 reflects an intermediate level of overall humanistic care competency among the study participants. The highest mean score on the U dimension suggests that these physicians possess comparatively strong abilities to recognize and empathically understand patient needs. This may be attributable to the emphasis placed on patient assessment and clinical communication in medical curricula. Through routine training and clinical practice, SRT and refresher doctor physicians accumulate considerable exposure to patient interactions, which cultivates their capacity to identify and respond to patient needs (10, 11).

In contrast, the relatively lower scores on the C and P dimensions indicate potential room for improvement. This also highlights prominent issues in clinical practice: physicians exhibit insufficient willingness and capability to proactively address complex humanistic care scenarios such as fetal anomaly disclosure and excessive maternal anxiety. Meanwhile, the high workload and cumbersome procedures of prenatal ultrasound examinations make it difficult for physicians to maintain long - term attention to patient needs and provide consistent responses. This situation suggests that targeted training focusing on courage and patience should be implemented in clinical practice to compensate for deficiencies in humanistic care competencies.

### 4.2 The core promoting effect of humanistic learning and training on humanistic care ability and suggestions for clinical implementation

The observed differences in CAI scores according to the frequency of humanities-related training during schooling and the frequency of active self-directed learning are notable. Physicians who received ≥3 sessions of humanities-related training demonstrated significantly higher humanistic care competency, underscoring the important role of systematic humanities training in fostering humanistic literacy. Formal humanities training broadens physicians’ perspectives and deepens their understanding of patients’ psychological and social contexts, equipping them to adopt a more genuinely patient-centered approach (12).

Physicians who engaged in frequent active self-directed learning similarly achieved higher CAI scores, highlighting the positive contribution of autonomous learning to humanistic care development. Such self-directed engagement allows physicians to tailor their acquisition of humanistic care knowledge to their individual needs and interests, thereby fostering a more comprehensive and integrated humanistic care philosophy (13). Based on the practical experience of prenatal ultrasound standardized training and advanced studies, the following specific training optimization recommendations are proposed: 1. During the medical education phase, incorporate a specialized humanistic training module for obstetric ultrasound, including mandatory courses on pregnancy psychology, prenatal communication, and conflict resolution between physicians and patients, with training frequency no less than 3 sessions. Case analysis and scenario simulation should be employed to enhance training effectiveness. 2. During standardized training and advanced studies phases, establish a regular humanistic learning mechanism, conducting periodic case seminars on humanistic care to share clinical practical experiences such as communication regarding fetal abnormalities and counseling for anxious pregnant women. 3. Develop an integrated online-offline humanistic learning resource library covering medical ethics, physician-patient communication, and pregnancy psychology, encouraging physicians to utilize fragmented time for self-directed learning. Humanistic learning outcomes should be incorporated into the standardized training completion assessment indicators.

### 4.3 Practical value and training path of empathy in prenatal ultrasound clinical work

The positive correlations between JSE-HP scores and CAI scores across all dimensions are consistent with the established role of empathy as a core component of humanistic care. In the context of prenatal ultrasound examinations, a physician’s empathic capacity enables them to recognize and appreciate the anxieties and concerns of pregnant women regarding fetal health, the examination process, and its potential findings. SRT and refresher doctors with greater empathy are better positioned to perceive these concerns from the patient’s perspective, provide attentive reassurance and explanation, and thereby enhance patient satisfaction and trust (14).

These findings suggest that SRT and refresher doctor programs should explicitly incorporate training strategies aimed at cultivating empathic capacity, employing pedagogical methods such as case-based analysis and role-playing simulations to enable physicians to engage more authentically with the emotional dimensions of patient experience.

To cultivate empathy skills in standardized training and advanced training physicians, the following clinical approaches can be adopted: 1. Conduct specialized empathy training through role-playing simulations of common prenatal ultrasound clinical scenarios, such as informing about abnormal fetal indicators and alleviating anxiety during prenatal examinations for advanced maternal age patients, enabling physicians to experience patient emotions immersively. 2. Implement a “mentorship program” for empathy practice guidance, where SRT/ refresher doctors in clinical communication, providing real-time feedback on communication deficiencies and imparting empathetic communication techniques. 3. Encourage physicians to participate in public welfare activities related to maternal psychological support, increasing exposure to diverse types of pregnant women to enhance empathy perception and response capabilities.

### 4.4 The indirect safeguarding role of family support and social support in physicians’ humanistic care competence and clinical management strategies

This study found that both family support (APGAR) and social support (PSSS) serve as independent positive predictors of humanistic care competence, revealing the indirect safeguarding role of physicians’ external support systems in clinical humanistic practice. Prepartum ultrasound residents and advanced trainees face multiple pressures including heavy clinical workloads, intense academic demands, and high specialized skill requirements. Strong family support provides emotional comfort and livelihood security, alleviating work and study stress. Social support elements such as recognition from colleagues and supervisors, as well as peer experience sharing, enhance physicians’ professional belongingness and work motivation. Only when physicians themselves experience support and care can they effectively convey warmth and patience to patients, thereby practicing humanistic care in clinical practice. These findings align with those reported by Yang et al. (15) who demonstrated that SRT and refresher doctor medical students with a more supportive emotional environment exhibited stronger humanistic care competency, thereby providing independent corroboration of the present results.

Based on this, medical institutions and residency training management departments should establish a multi-level support system for physicians: 1. Pay attention to the psychological state of residents and visiting physicians, establish regular psychological counseling mechanisms to alleviate their work and study stress; 2. Create a harmonious departmental working atmosphere, encourage mutual assistance and communication among colleagues, reduce internal work friction, and enhance team cohesion; 3. Improve the support system for residents and visiting physicians, arrange work schedules reasonably to avoid excessive overtime, ensure adequate rest time, and enable physicians to perform clinical work in good physical and mental condition.

The identification of frequent active self-directed learning of humanistic care knowledge as an independent predictor highlights the value of intrinsic motivation in professional development. During the SRT and refresher doctor period, physicians contend with extensive clinical responsibilities; nonetheless, proactive engagement with humanistic care knowledge reflects a commitment to holistic professional growth (16). To sustain and amplify this motivation, hospitals and SRT and refresher doctor management departments should provide diverse and accessible learning resources—including humanities-related textbooks, online courses, and academic lectures—and establish recognition and incentive mechanisms that reward exemplary engagement in humanistic care, thereby encouraging broader participation (17). The independent contribution of receiving ≥3 sessions of humanities-related training during schooling further supports the case for increasing the proportion and frequency of humanities content within medical education. Training curricula should encompass medical ethics, doctor-patient communication skills, and psychological principles, and be delivered through diverse modalities such as thematic lectures, small-group discussions, case analyses, and simulated scenario exercises to maximize learning effectiveness. Critically, training should be systematic, longitudinal, and developmentally scaffolded-commencing in the early phases of medical education and progressively deepening to ensure that physicians receive appropriately tailored humanities instruction at each stage of their training, thereby consolidating their humanistic care consciousness and competency over time (18).

This study found that the frequency of active and autonomous learning of humanistic care knowledge, as an independent variable, had the strongest positive predictive effect on humanistic care competence, as a dependent variable (*β* = 0.322). The present findings collectively demonstrate that humanistic care competency is the product of multiple interacting factors; accordingly, SRT and refresher doctor programs should adopt a comprehensive, multi-dimensional approach rather than focusing narrowly on any single factor. Alongside the cultivation of empathic capacity, physicians should be encouraged to attend to patients’ family and social backgrounds and to facilitate patients’ access to available social support resources. Hospitals may further consider organizing interdisciplinary training and exchange activities, fostering collaboration between prenatal ultrasound physicians and specialists in psychology and sociology to broaden interdisciplinary perspectives and enhance problem-solving capacity. This study still has certain limitations: Firstly, all participants were recruited from single-center medical institutions, which led to limited sample representativeness. As a result, it is difficult to directly generalize the findings to prenatal ultrasound physicians in different regions or at different hospital levels. Secondly, the use of self-administered questionnaires for data collection introduced potential subjective information bias, as it failed to integrate objective clinical behavior observations and patient satisfaction evaluations for comprehensive assessment. Thirdly, the study did not incorporate potential influencing factors such as physician personality traits, departmental team dynamics, or hospital humanistic development, resulting in insufficient exploration of factors affecting humanistic care capabilities. Future research can be conducted from three aspects: First, multicenter and large-sample studies should be carried out, including prenatal ultrasound standardized training and advanced training physicians from different regions and hospital levels to enhance the generalizability of research findings. Second, a comprehensive evaluation method combining “questionnaire surveys + clinical behavior observation + patient satisfaction assessment” should be adopted to reduce subjective bias and more objectively reflect physicians’ humanistic care capabilities. Third, further exploration should be conducted on the impact of personality traits, team atmosphere, and hospital humanistic development on humanistic care capabilities, aiming to construct a more comprehensive influence factor model for humanistic care capabilities.

## 5 Conclusion

In conclusion, the ability of SRT and refresher doctors in prenatal ultrasound to provide humanistic care is influenced by multiple factors, including humanities training, empathy skills, family support, and social support. These factors are closely related to clinical practice, medical education, and healthcare institution management.

Humanistic care is a vital component of prenatal ultrasound medical services. It not only impacts pregnant women’s healthcare experience and the harmony of the doctor-patient relationship but also indirectly enhances the quality of prenatal ultrasound diagnosis.

For clinical practice and medical education, the core insights are as follows: It is essential to establish a humanistic care competency training system that integrates “systematic training for medical students during school years + targeted standardized training + continuous clinical self-directed learning,” while also considering the development of external support systems for physicians. Efforts should be made from multiple dimensions, including learning and training, clinical practice, and psychological support, to enhance the humanistic care capabilities of prenatal ultrasound standardized training and advanced training physicians.

Ultimately, by improving physicians’ humanistic care competencies, prenatal ultrasound services can evolve toward a model of “technical precision + humanistic warmth,” providing pregnant women with higher-quality and more compassionate prenatal ultrasound medical services, thereby effectively enhancing the quality of prenatal medical care and patient satisfaction.

## Data Availability

The raw data supporting the conclusions of this article will be made available by the authors without undue reservation.
